# Direct Observation of Sophorolipid Micelle Docking in Model Membranes and Cells by Single Particle Studies Reveals Optimal Fusion Conditions

**DOI:** 10.3390/biom10091291

**Published:** 2020-09-07

**Authors:** Pradeep Kumar Singh, Søren S.-R. Bohr, Nikos S. Hatzakis

**Affiliations:** 1 Department of Chemistry & Nanoscience Center, University of Copenhagen, Thorvaldsensvej 40, C 1871 Frederiksberg, Denmark; pksingh@uakron.edu (P.K.S.); soeren@chem.ku.dk (S.S.-R.B.); 2 Department of Chemistry, University of Akron, Akron, OH 44325, USA; 3 Novo Nordisk Center for Protein Research (CPR), University of Copenhagen, Blegdamsvej 3B, 2200 Copenhagen, Denmark

**Keywords:** Sophorolipids, Single-particle measurement, liposomes micelles, anticancer, model membrane

## Abstract

Sophorolipids (SLs) are naturally produced glycolipids that acts as drug delivery for a spectrum of biomedical applications, including as an antibacterial antifungal and anticancer agent, where they induce apoptosis selectively in cancerous cells. Despite their utility, the mechanisms underlying their membrane interactions, and consequently cell entry, remains unknown. Here, we combined a single liposome assay to observe directly and quantify the kinetics of interaction of SL micelles with model membrane systems, and single particle studies on live cells to record their interaction with cell membranes and their cytotoxicity. Our single particle readouts revealed several repetitive docking events on individual liposomes and quantified how pH and membrane charges, which are known to vary in cancer cells, affect the docking of SL micelles on model membranes. Docking of sophorolipids micelles was found to be optimal at pH 6.5 and for membranes with −5% negatively charge lipids. Single particle studies on mammalian cells reveled a two-fold increased interaction on Hela cells as compared to HEK-293 cells. This is in line with our cell viability readouts recording an approximate two-fold increased cytotoxicity by SLs interactions for Hela cells as compared to HEK-293 cells. The combined in vitro and cell assays thus support the increased cytotoxicity of SLs on cancer cells to originate from optimal charge and pH interactions between membranes and SL assemblies. We anticipate studies combining quantitative single particle studies on model membranes and live cell may reveal hitherto unknown molecular insights on the interactions of sophorolipid and additional nanocarriers mechanism.

## 1. Introduction

Sophorolipids (SLs) are glycolipids naturally produced by Candida bombicola [1]. They are amphiphiles composed of a sophorose disaccharide coupled to long fatty acid and they exist in equilibrium between an acidic and lactonic form [2]. They are an eco-friendly and biocompatible class of amphiphilic biosurfactants that are used in a wide spectrum of medicinal application ranging from biosurfactant, cosmetic and detergent development [3]. Over the last years biosurfactants in general and SLs specifically have been used in biomedical sciences for their antifungal [4], antibacterial [5,6] anti-HIV [7], and anticancer [8,9] properties. The advantage of using SLs against cancer cells lines is their low side effect on normal tissue [10]. Therefore, SLs are widely appreciated as an efficient anticancer agent with verified effect in cell lines, i.e., MCF-7 and MDA MB-231 [10,11,12], cervical cancer cells [13,14], HT29, HT115, HCT116, esophageal cancer cells [15], human glioma cell line LN-229 [16] and Caco2, in addition to CCD841 colonic epithelium and MRC5 lung fibroblasts [17]. The precise effect of SL appears to vary between different cell lines [11] and micelles in lactonic form appear toxic in HEK293 cells. Cytotoxic effects of SLs produced by Wickerhamiella domercqiae show excellent results against esophageal cancer KYSE109, liver cancer H7402, and human lung cancer A549 [18]. Recently, SL were shown [15,19,20] to be a turn-on for apoptosis in cervical cancer cells. Apoptosis-inducing activities of SLs against liver cancer H7402 marked by morphological changes, i.e., membrane blebbing, cell shrinkage, and chromosomes condensation [19]. It also shows promising cytotoxic behavior against lung adenocarcinoma A549 and hepatocellular carcinoma HepG2 by inhibition of histone deacetylase and urokinase activities, respectively [21]. Earlier, SLs were used as excellent stabilizing agents in nanoparticle synthesis [22,23,24] while subsequently facilitating entry into the cell through the phospholipid membrane. A selective derivative of alkyl esters was applied in SLs production by Candida bombicola to enhance the cytotoxic effect against various cancer cell lines. Likewise, the chemoenzymatic alteration of SLs has also been applied to achieve greater biological applications [25]. Despite their emerging medicinal and industrial applications and cytotoxicity towards cancer cells [12], the mechanism underlying interactions with the cellular membrane and subsequent entry are not fully understood.

**Figure 1:**
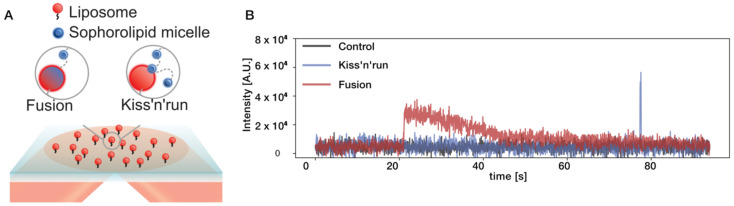
A fluorescent-based single particle assay to measure selective sophorolipid (SL) micelles interactions with liposomes tethered on passivated surfaces. (**A**) Cartoon representation of the single liposome assay to directly observe distinct and successive SL micelle docking event on liposomes. Biotinylated liposomes of different diameters are labeled with ATTO-655 and immobilized on a streptavidin-functionalized surface. The SL micelles labeled with a second chromophore (DiO-488) are added to the solution and allowed to interact with surface-tethered liposomes. Parallel imaging of the two different channels is acquired with a Total Internal Reflection Fluorescence (TIRF) microscope. The background corrected fluorescence intensity obtained from the red channel is used to quantify liposome spatial localization and observe directly the interaction of SL micelles in the blue channel. (**B**) Typical trajectories of SL micelle interaction on liposomes. Red color represents the successful docking of SL micelles on a liposome followed by chromophore photobleaching. The blue color represents kiss-and-run events where micelles transiently interacts with a liposome. A fraction of liposomes on the surface does not show any docking events; therefore, the trace displays the constant intensity over time. Similar profile traces (represents by Black color) were observed for non-fluorescently labeled SLs, liposomes labeled with DiO or DiO alone.

In this study, we firstly employ quantitative single particle microscopy [26,27], and arrays of surface tethered liposomes as model cell membranes [28,29] to observe directly several successive docking events of SL assemblies on individual nanoscale liposomes (see Figure 1) and provide a quantitative understanding of how membrane charge and acidic pH may regulate these interactions. Utilizing a biophysical system such as liposomes enables a deconvolution of regulatory effects (i.e., pH and surface charge) as well as the observation of several successive events that remain masked in contemporary measurements and thus provide insights into the underlying mechanism of interactions. We secondly perform confocal microscopy studies to observe interactions of SL assemblies with individual cells of commonly used cell models for healthy (HEK-293 [11]) and cancer (Hela [13,20]) cells, and thus, evaluate the in vitro measurements. Our quantitative analysis confirms an approximate two-fold increased interaction with the acidic pH membranes of Hela cells as compared to HEK-293 cell membranes. Concurrent with these results, our cell viability assay revealed an approximate two-fold increased cytotoxicity of SLs towards Hela cells as compared to HEK-293. By deciphering why SLs are more effective against cancer cells as compared to healthy cells, we provide mechanistic insights and a better understanding of SLs as potential therapeutics and/or drug careers for future cancer treatment.

## 2. Materials and Methods

### 2.1. Materials

All used chemicals were of analytical grade and purchased from Sigma-Aldrich (Copenhagen, Denmark) unless otherwise stated. DIO-488 chromophore was purchased from Thermo Fisher Scientific (Copenhagen, Denmark). Labeled phospholipids, 1,2-Dioleoyl-sn-glycerol-3- phosphoethanolamine-ATTO655 (DOPE-ATTO655) were purchased from ATTO-TEC GmbH, Siegen, Germany, Novozymes. Dye Cellmask (Red) and NucGreen was purchased from Invitrogen (Carlsbad, CA, USA).

#### Reagents for Tissue Culture

Tissue culture plasticware was purchased from BD Biosciences, (San Jose, CA, USA). Dulbecco’s Modified Eagle’s Medium (DMEM) and Fetal Bovine Serum (FBS) were obtained from Sigma-Aldrich (St. Louis, MO, USA). Penicillin/streptomycin and L-glutamine were obtained from Thermo Fisher Scientific, Gibco BRL (Waltham, MA, USA).

### 2.2. Preparation of Compounds

#### 2.2.1. Production of Sophorolipids

A loopful of *Candida bombicola* ATCC 22214 cells were taken from slant and seed culture was developed by transferring it to 10 mL medium consisting of MGYP medium (Malt extract 0.3%, Glucose 2%, Yeast extract 0.3%, Peptone 0.5%) for 24 h at 30 °C with 180 rpm. Then, the seed culture was transferred to 40 mL flask for development of starter culture and incubated for 24 h at 30 °C with 180 rpm. The fermentative culture was further carried out by transferring into 200 mL of medium mentioned above in 1 L Erlenmeyer flask under the same condition.

Sophorolipid was prepared by the resting cell method of starter mentioned above culture. The cells were re-dispersed in a production medium containing 10% glucose supplemented with oleic acid (1 g/100 mL) as lipophilic substrate. Sophorolipid was formed as a brown and viscous liquid, which was found to settle at the bottom of the flask after 96 to 120 h of incubation. After the incubation period, the cells were separated from the broth by centrifugation at 5000 rpm, 10 °C for 20 min. The SL formed was extracted from the supernatant with ethyl acetate. For the ethyl acetate phase, anhydrous sodium sulfate was added for removal of residual water. It was then filtered, and ethyl acetate was removed under vacuum. Hexane wash has been given to remove residual oleic acid. Acidic sophorolipid was prepared by base hydrolysis as discussed by Rau et al. [30]. Lactonic sophorolipid was purified by column chromatography Methanol/Chloroform solvent system. Both forms of sophorolipids were characterized by FTIR, LCMS spectroscopy (Figures S3 and S4).

#### 2.2.2. Micelle Preparation and Purification

Natural sophorolipid (SLs) was prepared by mixing acidic (SL(A)) and lactonic sophorolipid (SL(L)) forms of sophorolipid with 28:72, respectively. SLs was added in water with CMC concentration and sonicated 15 min in water bath and finally kept overnight for stabilization micelles formation day before the microscopic measurements. For dye molecule (DiO-488, 5 µg) encapsulation, the samples were dissolved in 10 mL ethanol solution and sonicated for 5 min. After sonication, the vial was dried under constant N_2_ flow and subsequently kept under high vacuum pressure for two hours. One milliliter pf buffer solution was added in a 1.5 mL Eppendorf tube, and kept for bath sonication for 15 min. Samples were incubated overnight before the experiment to allow micelles formation. The final solution was full of SLs+DiO-488 micelles, which were ready to use for Single-molecule study through Total Internal Reflection Fluorescence (TIRF) microscopy. The resulting sample was characterized using different modern analytical tools to know their morphology and utility.

#### 2.2.3. Liposomes Preparation

Liposomes were prepared by freeze-and-thaw method as we did recently [26,31,32]. Briefly, after adding the lipid to vial, it was kept under nitrogen flow for 10 min to remove all solvent, and finally, kept in high vacuum for at least one hour. Afterwards, the lipid film was rehydrated with selected buffer solution. The solution was then vortexed for 30 s and the solution was incubated for 30 min. Finally, the buffer solution was extruded (400 nm) and freeze-thawed 10 times at −45 to +45 °C in a water bath.

### 2.3. Data Acquisition

#### 2.3.1. Data Acquisition for Single Liposome Experiments

Micelles docking on liposomes. All single-particle measurement experiments were recorded on a Total Internal Reflection Fluorescence (TIRF) microscope (IX 83, Olympus, Tokyo, Japan). Images were captured using two EMCCD cameras (Image X2, Hamamatsu, Hamamatsu, Japan) and an oil immersion 100× objective (UAPON 100XOTIRF). DiO-488 and ATTO-655 fluorophores were excited using 488 nm and 640 nm solid-state laser lines, respectively (Cellsens, Olympus). Imaging of liposomes (ATTO-655) was done using 50 ms of exposure time, 300 EM gain. All experiments were done using the same experimental setup. Briefly, liposomes was tethered to the glass support as described earlier [26,31,32] and visualized by exposing them with a 640 nm laser beam for 50 ms. Similar measurements were recorded with Blue laser 488 to make sure that the background of the experiment was free of crosstalk. Once the liposomes were imaged, SL micelles solution with encapsulated Chromophore (DiO-488) was flown into the sample by continuous flow while recording their interaction under a 488 nm laser beam at a concentration of 0.12 mg/mL (allowing giving optimal docking without disrupting liposomal membranes). Minor variations from pipetting errors were averaged from repeated experiments. The concertation used was similar to the concentration reported to be cytotoxic in HEK-293 lines for SL, albeit in the lactonic form [11]. We have collected 2000 frames with 50 ms exposure time with Blue laser over 1.43 min experiment. Similar sets of experiments were performed over all three different pH buffer liposomes. All the data were collected and analyzed by a homemade python script adapted from an earlier publications by our group [31,32,33]. The interactions of SL micelles were recorded as docking and kiss-and-run type events on the liposomes.

#### 2.3.2. Data Acquisition of CLSM Images

All CLSM images were acquired using an Olympus confocal microscope (IX83) and an UPLSAPO 100x (NA 1.40) oil emersion objective. For quantification of the SL uptake, cell membranes were stained using CellMask Deep Red Plasma Membrane Stain (ThermoFisher, Waltham, MA, USA) and SL micelles with DiO-488, using the 488 and 635 laser lines at 7% and 10% power, respectively. For both conditions, the photomultiplier tubes (PMT) voltage was set to 610, offset 10 and gain 3, while images were acquired with a 2 µs scan time per pixel. For the cell viability assay, the cell membrane was stained using CellMask Deep Red Plasma Membrane Stain (ThermoFisher) and dead cells using NucGreen marker (ThermoFisher), using the 635 and 488 laser lines at 7% and 6% power, respectively; images were recorded with a 2 µs scan time per pixel. For both conditions, cells were incubated for 15 min with the membrane stained, and subsequently washed three times using PBS buffer. Images were then normalized as follows to exclude bias from variations in local density of particles:

Intensity inside cells/Cell area * 1/Total image intensity (1)

### 2.4. Live Cell Experiments

#### 2.4.1. Cell Culture

The cells were grown in DMEM containing 2 mM L-glutamine supplemented with 10% fetal bovine serum and 100 U/mL of penicillin-streptomycin. The cells were incubated in a humidified 5% CO_2_ incubator at 37 °C. The cell viability was determined by Cellmask dye uptake as described below. Briefly, the cells were seeded at a density of 1 × 10^5^ cells/mL density in 8-well plates. An untreated group was kept as a negative control. The cells were treated with similar amount of sophorolipids (0.12 mg/mL) used for the single molecule measurement for 30 min followed by washing three times with PBS buffer. All incubation with SLs was done in full growth media as described above.

#### 2.4.2. Microscopy Cell Viability

The cell viability was analyzed by recording the number of live cells present after 1 h and 18 h of incubation using a confocal microscopy (IX81, Olympus, Tokyo, Japan ). For cytotoxicity, Cellmask red marker was applied to the counting of live cells, while the NucGreen marker (ThermoFisher, membrane disruption dye) was used for the number of dead cells. All cells were outlined using a custom made routine in python [33] and afterwards counted manually. All the experiments were performed in triplicates and repeated twice, and the data are presented as mean ± SD. To measure the degree of apoptosis and necrosis, the widely used GFP-CERTIFIED^®^ Apoptosis/Necrosis Detection System (Enzo life sciences, New York, NY, USA) [34,35] was used together with CellMask Green Plasma Membrane Stain for cell counting. All the experiments were performed in triplicates. Statistical comparison was done using a two-sided Welch’s *t*-test to determine if the observed differences are significant (asterisks represent the following *p*-values: * < 0.01, ** < 0.001, *** < 0.0001). All incubation with SLs was done in full growth media as described above. Prior to any imaging, cells were carefully washed three times with PBS buffer. The percentage cytotoxicity/necrosis/apoptosis was calculated as:

% cytotoxicity/necrosis/apoptosis = (responsive cells)/(total cells imaged) × 100%(2)

#### 2.4.3. 3-(4,5-Dimethylthiazol-2-yl)-2,5-Diphenyltetrazolium Bromide (MTT) Cell Viability

MTT cell viability was done as previously described [36]. Shortly cells were seeded in 96-well culture plates at a seeding density of approximately 2 × 10^5^ cells per well and grown for 24 h as described above. After 24 h, the media was carefully replaced with media containing the desired concentration of SLs (0.12 mg/mL) and incubated for either 18 h or 1 h. Hereafter, MTT in PBS was added to a final concentration of 0.8 mg/mL and incubated for 4 h. Following the final incubation, the media was carefully removed and 200 µL DMSO was added to each well. After 20 min of incubation in the dark, the absorbance was measured at 570 nm.

### 2.5. Data Analysis

#### 2.5.1. Data Analysis for Single Liposome Experiments

Extraction of background corrected intensities for all liposomes was done using the earlier published method in Python [31,37]. Subsequently, the detection of interaction events was quantified using an adapted version of earlier mentioned software. Here, events lasting only few frames would be characterized as kiss-and-run type events and thus distinguishable from long lasting events. Additional traces can be seen in Figure S6.

#### 2.5.2. Exponential Fitting to Docking Rates

**Figure 2:**
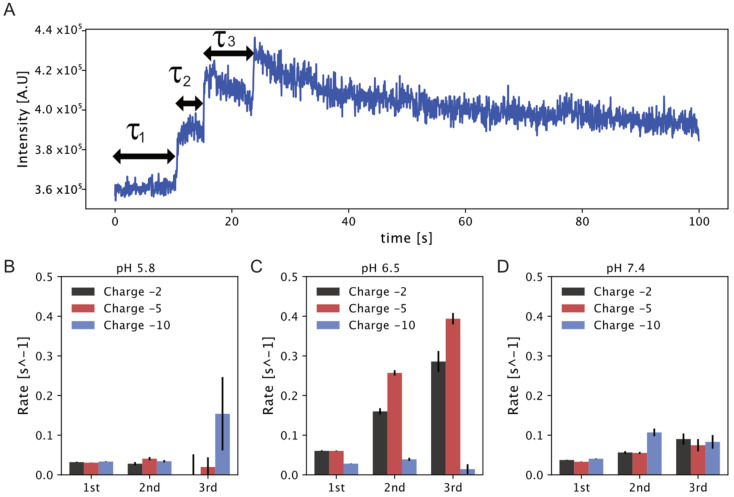
Direct observation of several successive interaction events, quantification of their frequency, docking times, and their dependence on initial liposome charge and solution pH. (**A**) Representative trace displaying multiple successive docking event on individual liposomes data of pH 6.5 liposomes with 5% negatively charged lipids (see SI for additional traces). (**B**) Docking rate for pH 5.8 liposomes on liposomes with varying negative charge. (**C**,**D**) represent the docking rate for pH 6.5 and pH 7.4, respectively. Data from >200 liposomes per condition (see Table 1). Error bars represent one standard deviation.

To evaluate the synergistic effect of multiple SL micelle dockings on liposomes, we extracted the waiting time *τ* between consecutive events (see Figure 2). As expected, this followed a single exponential decay, and thus, we fitted the waiting times using an unbinned likelihood fitting scheme as we did recently [37]. All data were fitted to the equation:

(3) $$N(t)=A*e^{-\lambda *\tau }$$

where *A* is the scaling factor and *t* is the observed waiting times (see Figure S8 for representative fit).

#### 2.5.3. Quantification of CLSM Images

To quantify the combined uptake and membrane interacting SL micelles on live cells, we used a slightly modified version of earlier published software [31,33,37]. Briefly, based on the membrane signal, the software will create a binary mask for all cells and use this to integrate fluorescent intensity from the cell interior (including the membrane) and exterior, respectively. This would then be normalized to the cell area and total image intensity to avoid bias. Statistical comparison was done using a two-sided Welch’s *t*-test to determine if the observed differences are significant (stars represents *p*-value of: * < 0.01, ** < 0.001, *** < 0.0001).

## 3. Results

### 3.1. Sophorolipid Micelle Docking on Model Membranes

To analyze the SL micelle interaction with membranes, we utilized arrays of liposomes tethered on passivated surface at low surface densities using streptavidin biotin interactions. Imaging was performed by Total Internal Reflection Fluorescence microscopy (TIRFm), which allowed us to monitor in parallel 200–300 liposomes of different diameters per frame [26,31]. Dimensions of nanosized liposomes appear as diffraction-limited spots by optical microscope and it cannot be directly analyzed from a recorded image. We recently showed that the integrated background corrected fluorescence intensity of liposomes with labeled lipids is proportional to the square root of the liposome diameter [26,31]. Labeling liposomes with Atto-655-DOPE allowed us to both extract the nanoscale dimensions with high accuracy (±5 nm) and identify their sub-resolution spatial localization [37,38]. We prepared SL micelles by sonication driven force and characterized by dynamic light scattering (DLS, Figure S5) and zeta-potential (−17.30 mV, see Figure S6). Implementing a chromophore in SL micelles allowed us to observe their interaction with the membranes of liposomes in real time.

In a typical experiment we tethered liposomes containing 0.5% Atto-655-DOPE, with the desired lipid composition (see Supplementary Table S1 for liposome composition) on a microscope glass surface passivated with PLL-PEG. Subsequently, the solution of SL micelles loaded with DiO-488 was flowed in using a flow cell device as described recently [31]. Parallel recording of both the 655 nm and 488 nm channel, for a total of 2000 frames with 50 ms exposure allowed us to observe directly the docking events of SLs on individual liposomes (see Figure 1 and Figure S7 for more data). Recording on the 488 nm channel prior to SL addition allowed correction for minimal cross talk between the channels. The high fidelity of the single particle readout allowed us to observe both transient and long-lasting interactions of micelles with individual liposomes (blue and red trace in Figure 1, respectively). We refer to transient interactions lasting less than two frames as kiss-and-run events. In most cases, the interaction of SLs with model membrane was persistent over long periods of time and resulted in the eventual bleaching of DiO-488 (red trace in Figure 1). This prolonged interaction would result to their fusion to the membrane as SLs have been shown to proficiently fuse with membrane [39]. Quantitative image analysis allowed us to observe directly several successive docking events and quantify their abundance and frequency.

To ensure precise measuring the properties of SL micelle interactions on liposomes, we performed several control experiments. Firstly, we ensured that docking events are not individual DiO molecules or DiO assemblies that are formed in the experimental conditions. To confirm, we added to surface tethered liposomes a mixture of DiO-488 in DMSO at the same concentration (5 ng) as used for SL mixtures. We recorded an overall enhanced background signals with immobilized liposomes but not distinct docking events (see Figure S7). This, in conjunction with the exponential decay of the intensity upon docking (Figure 1B), confirms that interactions are mediated through SL assembly and does not originate from docking of individual freely diffusing chromophores. To ensure that SL interaction does not alter chromophore properties resulting in false positives, we performed identical measurements with SL micelles that were not labeled, but could otherwise interact with membranes. The absence of signal confirms the validity of our assay (see Figure S7). We next prepared 1,2-dioleoyl-sn-glycero-3-phosphocholine (DOPC) liposomes labeled with DiO and allowed them to interact with the surface tethered liposomes. No docking events was found, thus confirming that SL micelles is the driving force for the assembly (see Figure S7).

The parallelized readout on hundreds of individual liposomes per field of view allowed us to observe directly several successive interaction events on individual liposomes. In each event, SL micelles dock and interact irreversibly with the liposome as shown in Figure 2 for three subsequent events (see Figure S7 for additional traces). The docking of each SL micelle will result in a stepwise increase of the signal, and the docking of multiple micelles results in multiple steps as shown in Figure 2. Prolonged imaging will bleach the DiO chromophore, as shown by the mono-exponential decay of intensity, also confirming that each docking event is an SL assembly rather than individual DiO molecules (see Figure 1B, red trace, and Figure S7). Quantification of interaction events and their separation from kiss-and-run type of events was done using custom made script in Python, based on earlier publications [31,33,37]. Briefly, events were detected using a method similar to earlier published [31,37,40,41], where each intensity trace is scanned for changes larger than five standard deviations. Once detected, the length of micelle interactions were categorized using the first derivative and labeled as either kiss-and-run if there are fewer than two frames or else as a normal event. A total of >14,000 liposomes were imaged across all experimental conditions, out of which we found more than 12,000 and 12,500 events and kiss-and-runs, respectively (see Table 1 and Supplementary Table S2 for detailed statistics).

We hypothesized that earlier reported SL interactions with cancer cells are mediated by the cells acidic pH and increased charges, which originates from the increased glycocalyx [42] (i.e., Hyaluronic acid percentage on the cancerous cell membrane in comparison to the normal one). To confirm, we quantified the SL micelle interactions with liposome model membranes, systematically altering their pH and negatively charged surface. We varied the membrane charge by constructing liposomes with (2%, 5%, and 10% (mol:mol)) phosphatidylserine (DOPS) content (see Supplementary Table S1 for detailed liposome composition and liposome zeta potential). Similar to the examined effect of pH, each membrane charge was tested at normal pH 7.4 [43], pH 6.5 (similar to the extracellular pH around cancer cell membrane [43,44]), and highly acidic pH 5.6.

We quantified the effect of pH and model membrane charge on the abundance and number of successive dockings as well as the kiss-and-run interactions of SLs with model membranes (see Table 1 and Supplementary Table S2). At pH 6.5, the fraction of events per liposome displayed a significant dependence on charge and at 5% charges was found to be 1.57 events per liposome. This is higher than in all other conditions, where they are practically all below 1 and show minute, if at all, dependence on charges and pH. This optimal “sweet-spot” is further supported by attaining the highest percentage of liposomes with multiple events (52%), with all other conditions displaying between 30% and 40%. Experiments at pH 5.8 showed a relatively constant fraction of docking events per liposome, independent of liposome charge density (0.73, 0.72, and 0.66% docking for −2%, −5%, and −10% surface charge, respectively). The likelihood of more than one event was also found to be <0.36%. The fraction of docking events per liposome was higher at pH 7.4 (0.97, 1.04 and 0.83% docking for −2%, −5% and −10% surface charge respectively), while the likelihood of more than one event was slightly increased, but in both cases showed no dependency on charges. The fact that we observe more than 1000 events for almost all conditions further supports that this is not an artifact of the experiments, but rather an inherent feature of the system. The summarized results in Table 1 display that a) altering the charge density and pH does indeed modulate micelle docking efficiency and b) optimal docking conditions seems to be at pH 6.5 and 5% negative charge. The recorded optimal docking conditions are in accordance with the acidic pH of cancerous environment and may provide clues on the specificity of SLs towards target cancer cells over healthy cells.

The high-fidelity readout of the single particle assay allowed us to quantify the waiting time between each of the consecutive SL interaction event with individual liposomes shown as τ_1_ to τ_3_ in Figure 2. By fitting the waiting time between events using an exponential decay, we were able to extract the docking rate for multiple events and reveal exactly how pH and charge affect fusion (see the supporting information (SI) for exponential fits Figure S7). The fact that the absolute number of events displays only a minute dependence on variations in pH and charge confirms that reported rates are not due to SL micelle concentration variations (see Table 1). Figure 2B–D represents the docking rate for multiple consecutive events at the three different liposome surface charge for all tested pH values. At pH 6.5, docking rates show a significant increase for subsequent docking evens. For the first event, the rates at −2% and −5% charges are practically identical and doubled as compared to the rates at pH 5.8 (0.06 s^−1^ as compared to 0.032 s^−1^). The second event occurs three and four times faster than the first event at −2% and −5% charge, respectively, at pH 6.5 (0.06 s^−1^ and 0.06 s^−1^ as compared to 0.16 s^−1^ and 0.26 s^−1^). Similarly, the rate for the third event is amplified even further and increased approximately four and eight times as compared to the first event (0.06 s^−1^ and 0.06 s^−1^ as compared to 0.28 s^−1^ and 0.39 s^−1^), suggesting the existence of a positive feedback loop type of mechanism. Interestingly, the rates at −10% charges on the other hand are similar to the rates at pH 5.5 and independently of sequence of events. Charges for the optimal interaction at pH 6.5 appears to be −5%. The overall increased rate for subsequent events indicates some form of synergistic effect where docking of SL lipids could destabilize the membrane, or reduce the charge density, allowing the faster docking and fusion of subsequently SL. This effect appears to be alleviated at elevated membrane charges. The fact that we recorded a positive feedback for subsequent events also supports that SL docking leads to successful fusion.

In the case of pH 5.8, the rate of reaction for the first and second consecutive docking event was almost comparable for each sample independent of membrane charges (−2%, −5%, and −10% charge liposomes). The rate of reaction for the third docking event appeared higher for −10% charged liposomes (with large error bar) though it remains independent of the event number and very low for −2% and −5% charged liposomes. Similarly, at pH 7.4 the rate of reaction for the first docking event showed practically identical rates of 0.04 s^−1^ at all charges and the second docking event resulted in minute increase for both −2% and −5% charges, while it gets doubled for liposomes with −10% charge. The rate for the third event here was around 0.08 s^−1^ and independent of liposomes charges. In summary, at pH 7.4, or the very acidic pH 5.5, there is a very small effect on micelles docking either for charges and/or subsequent events. On the contrary, at slightly acidic, pH 6.5, charges have a dominant role increasing the docking rates for multiple events. The recorded enhanced docking rate are in agreement with the known increased interaction of SL micelles with acidic cancerous cell membrane. Our findings, thus, could explain why SLs preferentially binds and fuses with cancer cell membrane and display enhanced cytotoxic effect for anticancer therapeutics, as discussed earlier. They also show that the docking of the first event acts as a positive feedback loop for the second docking event due to probably membrane destabilization.

### 3.2. Sophorolipid Micelle Docking on Mammalian Cells

#### 3.2.1. Sophorolipid uptake on Hela and HEK-293 Cells

**Figure 3:**
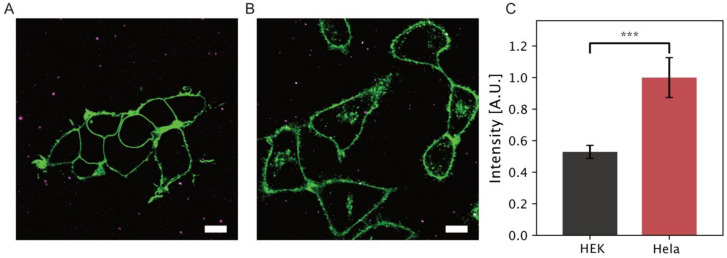
Two-color imaging displaying (**A**) HEK-293 cells and (**B**) Hela cells labeled with CellMask Red and SL micelles loaded with DiO. Scale bar 10 µm. (**C**) Quantification of total normalized intensity of DiO localized on the membrane or inside the cells. A two-fold increase of the DiO signal is observed with Hela cells, indicating a stronger interaction and higher internalization in Hela cell as compared to healthy HEK-293 counterparts. Data from 12 individual images per condition (three technical and two biological replicates resulting in six total repeats). Error bars corresponds to one standard deviation, (*** *p* < 0.0001).

We then evaluated our single liposomes in vitro findings by measuring the docking of SL micelles on two types of mammalian cell lines (Hela and HEK-293). We used Hela cells as a cancer cells model and HEK-293 as a healthy cell model, as they have been widely employed for SL studies [10,11,20]. Both were grown for 24 h in eight vial coated glass (see methods) chambered slides in a 5% CO_2_ incubator with 37 °C temperature before imaging using an inverted confocal microscope. Cell membranes were labeled with Cellmask Red membrane lipid marker. Data were recorded with and without (control sample) addition of an equal amount of SL micelles (50 µL sample, 0.12 mg/mL). Confocal microscopy images capture SL micelles (magenta pseudo-color) in solution and interaction with the cells. Figure 3 displays snapshots of the Sls DiO-micelles in solution and interacting with cells. Upon docking and membrane fusion, the resulting mixing of lipid species and rapid diffusion away from the docking spot will result in fluorescent signal dilution and loss [45,46] (see Figure S9 for time lapse of particle docking and subsequent signal loss). Analysis was performed in python using a custom routine adapted from [33,47]. Shortly, a binary mask was used to integrate the intensity of fluorescence signal localized with cell membrane and internalized or outside of the cell membrane (Figure 3). We created a mask based on the cell membrane fluorescent stain (thus also defining the total cell area) and collected all intensities inside cells and outside (see Section 2.3.2).

Plotting the background corrected fluorescent intensity of DiO localized on cell membrane and internalized for both cell lines reveals an almost two-fold increase intensity within the membrane boundaries for Hela cells as compared to HEK-293 cells. We note that SL micelles upon docking with membranes may fuse or be internalized intact via endocytosis pathways. While deciphering the mechanism of cell entry is beyond the scope of this study, integrating the overall fluorescent signal within the membrane boundaries accounts for internalized SL independently of the entry mechanism. Our cell data support our in vitro recording on model membrane and observed increased interactions with the acidic membranes of Hela cells. They also support earlier findings that sophorolipid encapsulations ensure that maximum amount therapeutics cross cell membrane barriers and attain reduce therapeutic index for an anticancer molecule [10,48].

#### 3.2.2. Cell Viability

To compare our model membrane outcome on a mammalian cell line, we evaluated the cytotoxicity of SL micelles on Hela cell lines by cell viability assay at 1 and 18 h of incubation, respectively. Sophorolipids are reported to be nontoxic for healthy cells [10] while they are expected to lead to cell death for cancer cell lines. Though the cytotoxicity of acidic SLs is negligible, some researchers mentioned its toxic behavior for HEK-293 cells [11]. The actual mechanism of the cytotoxic effect on cancer cells is not yet clear, but is anticipated to operate via inducing the apoptosis process. Here, we used NucGreen dead 488 dye solution, that stains the nuclei of cells with compromised plasma membrane integrity, to quantify the fraction of dead cell upon SL interactions. In a typical experiment, we first stained the cell membrane with Cell-Mask (Deep Red Plasma membrane Stain), so as to quantify the total number of cells present on the slide. Excess amount dye was washed by repeated washing three times with buffer solution and then NucGreen 488 solution was added dropwise and incubated for 10 min in the microscope chamber. As a control experiment on non-cancer lines we employed HEK-293. Experiments in both cases were performed in the presence and absence of SL addition to eliminate imaging artifacts, such as phototoxicity effects.

**Figure 4:**
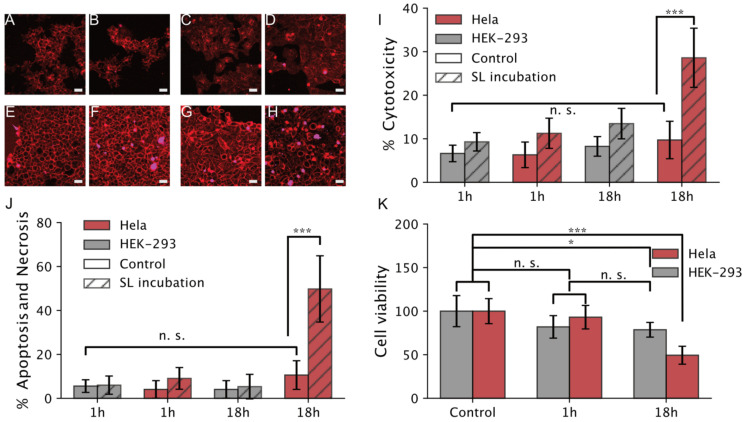
Cell viability assay for SL micelles and their effect on mammalian Hela and HEK-293 cell lines (scale bar 10 µm). (**A**,**B**) HEK-293 in control and SL micelles, respectively, after 1 h of incubation with SLs. (**C**,**D**) Hela cells in control and SL micelles, respectively, after 1 h of incubation. (**E**,**F**) HEK-293 cells in control and SL micelles, respectively, after 18 h of incubation. (**G**,**H**) Hela cells in control and SL micelles, respectively, after 18 h of incubation. The experiment was recorded under a confocal microscope at 1 h and 18 h of incubation with SL micelles. The control sample was kept with both cell lines to the measurement of SL micelles’ cytotoxic behavior. (**I**) Graphical representation of cytotoxicity obtained from the incubation of SL micelles with HEK-293 and Hela cells using NucGreen. Error bars correspond to one standard deviation. (**J**) Percentage of cells displaying late apoptosis or necrosis after incubation with SLs. (**K**) MTT cell viability after 1 h and 18 h of incubation with SLs. Error bars correspond to one standard deviation (error bars in all graphs are from two biological samples with three technical repetitions, resulting in six total repeats), (* *p* < 0.01, *** *p* < 0.0001).

We found SL incubation to strongly affect Hela cell viability Figure 4 after 18 h of incubation. We initially examined the effect of SL micelle incubation for 1 h. Comparison of the results of Hela cells with HEK-293 and controls without SL addition (Figure 4A–D) shows a minute difference between the cell lines, confirmed using a two-sided Welch’s t-test. We quantified 11.3% ± 3.5% Hela cell deaths upon SL incubation as compared to 6.31% ± 3% (*p*-value: 0.025) for no SL incubation, see Figure 4I for quantifications. HEK-293 cells, on the other hand, display the same within error cell deaths (9.3% ± 2.1% and 6.6% ± 1.9%) with and without SL incubation, respectively (*p*-value: 0.028). Increasing the SL incubation to 18 h, on the other hand, resulted to a remarkable increase of cell toxicity for Hela cells (28.6% ± 6.8%) as compared to no SL control (9.7% ± 4.3%, *p*-value < 0.001). In comparison the toxicity on HEK 293 cells was 2× lower 13.5% ± 3.5%, slightly higher than the non-SL control (8.25% ± 2.25). This is interesting, as recent studies with SL reported cytotoxicity for HEK-293 cells in similar concentration and longer incubation times, albeit they worked with the lactonic form [11]. Additional measurement reporting the fraction of cells undergoing late apoptosis, necrosis and a formazan based MTT assay confirmed the previous results, see Figure 4J,K, respectively. Here, Hela cells incubated for 18 h with SLs showed a significantly increase of necrosis 49.8% ± 15.1% (two-sided Welch’s t-test, *p*-value < 0.001), as compared to ~10% for the remaining conditions. We observe no significant effect after 1 h of incubation with SLs, with the data being inseparable from controls without SL. Similarly, no effect was observed for both controls and SL incubation in HEK-293 cells, confirming the results obtained using NucGreen. The proficiency of SLs targeting cancer cells specifically is further supported by a) the fact that the remaining conditions are both identical with error and b) an excellent agreement with the data represented in both Figure 4I,K for all conditions.

Interestingly, the MTT assay (see Figure 4K) shows minor cytotoxicity for HEK-293 that may originate from the lactonic form, as shown earlier [11]; however, the overall results are in great agreement with previously discussed viability assays thus confirming the SL preference for cancer cells.

In summary the cell viability assays display a 2× increased cell toxicity for cancer cell lines vs. normal cell lines. This is in line with our single particle assays reporting enhanced interaction and internalization of SL assemblies on the Hela cells. Probably, the high number of micelles docking and subsequent fusion induce higher cytotoxicity towards cancer cells. Our findings are in line with recent researcher work [10,48] reporting sophorolipid encapsulation reduces the therapeutic index of the anticancer drug molecule and lowers the required amount of dose for anticancer treatment.

## 4. Discussion

SLs are widely used for a spectrum of industrial application ranging from antibacterial agents to cosmetics, but also in various industrial applications such as biosurfactants and detergent development. Recently, their antifungal, antibacterial and importantly their efficacy as anticancer agents was found. However, the mechanism underlying its cell entry proficiency remain unclear. Here, we used single particle studies on model membranes, live cell imaging, and cytotoxicity assays to observe directly the SL membrane interaction and decipher the parameters underlying optimal docking. Our biophysical in vitro studies provide direct evidence that acidic pH (6.5) and charges (5%), like the one found cancerous cells, is a dominant feature underlying their interaction with membranes. Our direct observations of several successive docking events and quantification of the docking rates indicated a positive feedback loop driving the enhanced interaction of subsequent events. Our live cell imaging on Hella cancer cells lines confirmed these by recording a 2× fold increased interaction with cancer cell as compared to control HEK-293. This was further corroborated by cell viability assays showing at least the 2× increased cytotoxicity in cancer cell as compared to control cells with excellent agreement across orthogonal viability assays. Our data thus provide direct multivalent evidence that sophorolipid micelles have a higher affinity for the cancer cell environment in comparison to the healthy cells, providing clues on SL micelles supporting and synergistic role for anticancer treatment.

## 5. Conclusions

In summary, the combined single particle readouts on model membranes and cell studies here provides hitherto unknown insights into the role of pH and membrane charge role in the proficient docking of sophorolipid micelles towards model membranes. The quantitative insights are confirmed by single particle studies on live cells and cell viability assays, providing clues on the underlying mechanism behind their reported anti-cancer properties and the selective increased cytotoxicity to cancer lines. These findings may pave the way for wider use of SL or additional nanocarrier systems enhancing the bioavailability of their cargo for low dose prescription. Especially important for this is the understanding of nanocarriers interactions with mucosal layer around the cells. Quantitative single particle studies combined with live cell imaging will be crucial in the endeavor.

## Figures and Tables

**Table 1:** Sophorolipid (SL) micelle docking and kiss-and-run events data measured with different pH and charged model membrane. The table represents the total number of liposomes and subsequent docking on individual buffer pH (5.8, 6.5, 7.4) with negative charge (−2%, −5%, −10%). See Supplementary Table S2 for additional statistics.

pH	5.8			6.5			7.4		
SURFACE CHARGE	−2	−5	−10	−2	−5	−10	−2	−5	−10
LIPOSOMES	1242	1608	1817	2011	1214	1666	1399	1427	1711
TOTAL EVENTS	902	1164	1192	1388	1903	1315	1370	1495	1427
LIPOSOMES WITH EVENTS	845	1083	1093	1197	1244	1229	1195	1325	1309
KISS-AND-RUNS	537	1197	1192	1221	1040	1512	1891	2218	1911
EVENTS PER LIPOSOME	0.73	0.72	0.66	0.69	1.57	0.79	0.97	1.04	0.83
LIKELIHOOD OF MULTIPLE EVENTS	0.33	0.32	0.36	0.3	0.52	0.35	0.39	0.39	0.34

## Supplementary Materials

The following are available online at https://www.mdpi.com/2218-273X/10/9/1291/s1, Figure S1: Sketch diagram of model membrane design and SL micelle docking events, Figure S2: Protocol for the production of sophorolipids, Figure S3: FTIR spectra of sophorolipids, Figure S4: LCMS spectrum of acidic and lactonic sophorolipids, Figure S5: Dynamic Light scattering (DLS) data of sophorolipids micelles (red) and dye encapsulated sophorolipid micelles (black), Figure S6: Zeta potential of SL micelles, Figure S7: Additional traces and control experiments of SL micelle docking on surface tethered liposomes, Figure S8: Exponential fitting of consecutive rates, Figure S9: Time lapse of an SL micelle on Hela cell, Table S1: Individual liposome composition and zeta potential, Table S2: Statistics of all observations measured with three different pH values and three different surface charge.
