# Supplementary material for: Direct Observation of Sophorolipid Micelle Docking in Model Membranes and Cells by Single Particle Studies Reveals Optimal Fusion Conditions
Source: Biomolecules. 2020 Sep 7;10(9):1291. doi: 10.3390/biom10091291 (PMC7564020; doi:10.3390/biom10091291)
Supplement: Supplementary file 1 [file biomolecules-10-01291-s001.pdf]

Supplementary information

Direct Observation of Sophorolipid Micelle Docking in Model Membranes and Cells by Single Particle Studies Reveals Optimal Fusion Conditions

Pradeep Kumar Singh [1,2], Søren S.-R. Bohr [1,3] and Nikos S Hatzakis [1,3,*]

[1] *Department of Chemistry & Nanoscience Center, University of Copenhagen, Thorvaldsensvej 40, Frederiksberg C 1871, Denmark*; soeren@chem.ku.dk
[2] *Department of Chemistry, University of Akron, OH, USA*; pksncl@gmail.com
[3] *Novo Nordisk Center for Protein Research (CPR), University of Copenhagen, Blegdamsvej 3B, Copenhagen 2200, Denmark; hatzakis@chem.ku.dk*

* Correspondence: hatzakis@chem.ku.dk

The supporting information consist of 9 pages with a total of 9 figures and 2 table

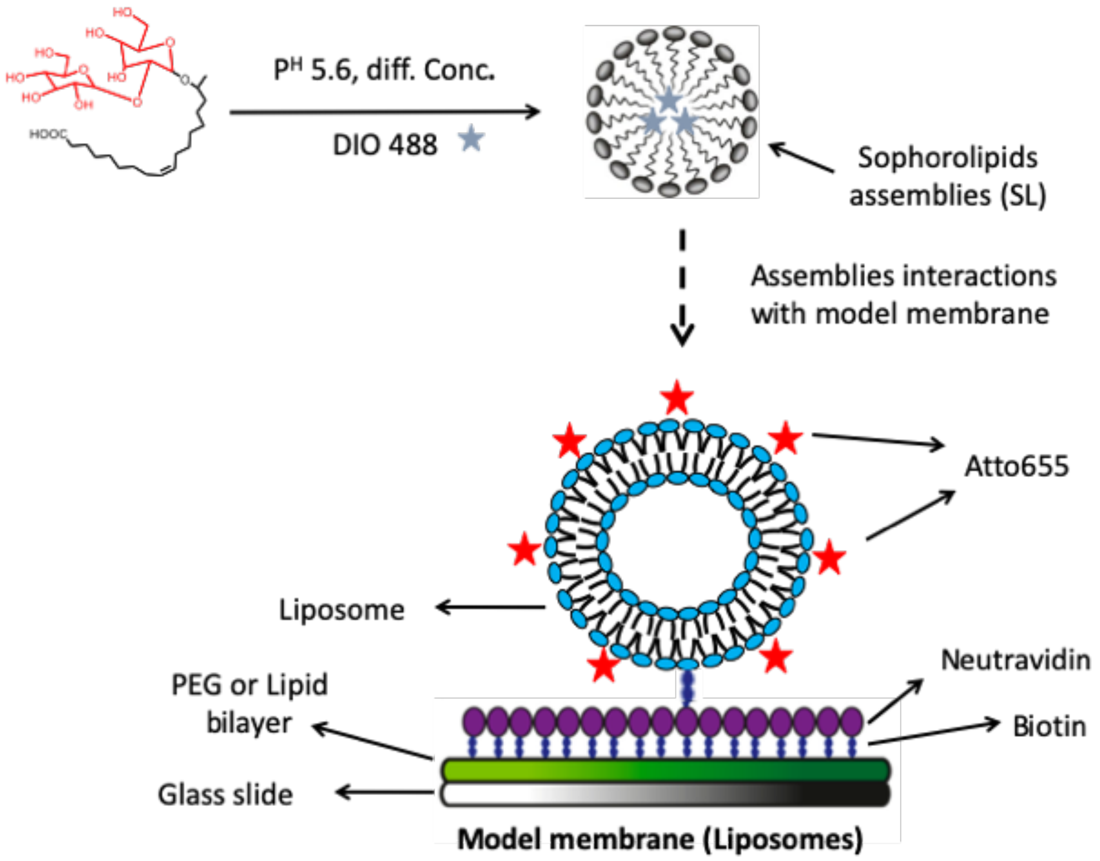

Figure S1. Sketch diagram of Model membrane design and SLs micelles docking events

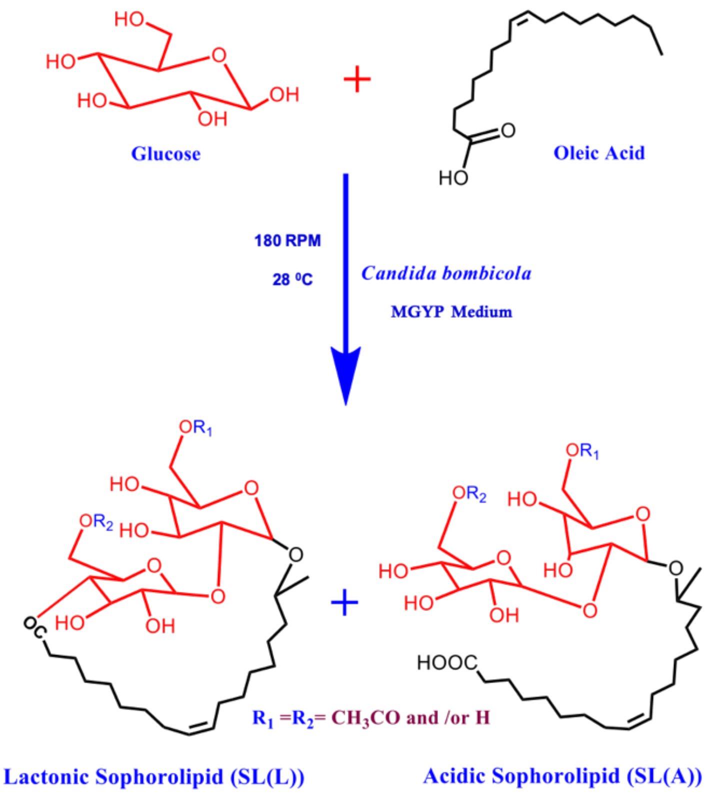

Figure S2. Protocol for the Production of sophorolipids

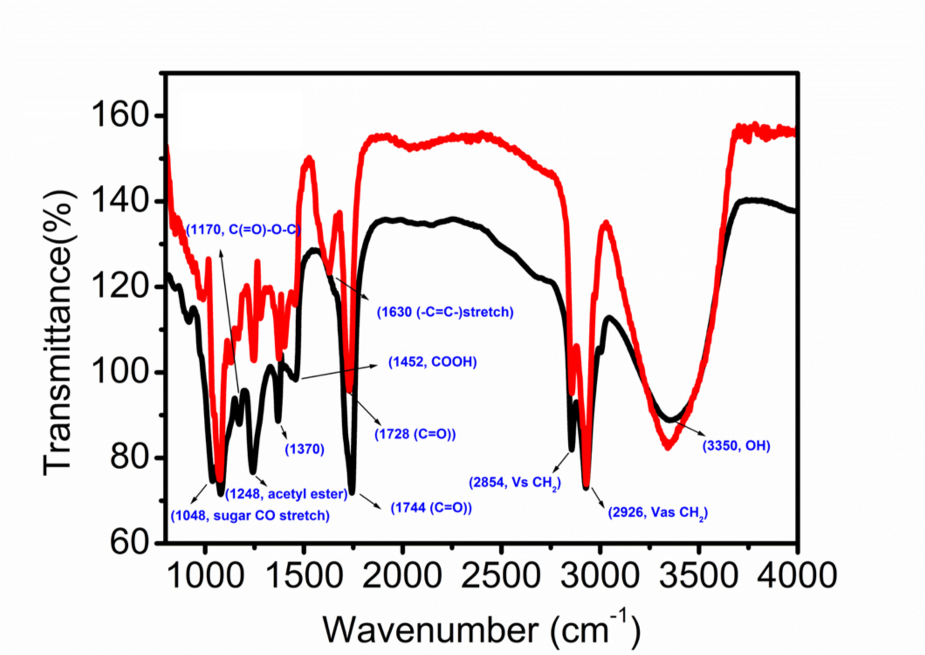

Figure S3. FTIR Spectra of Sophorolipids

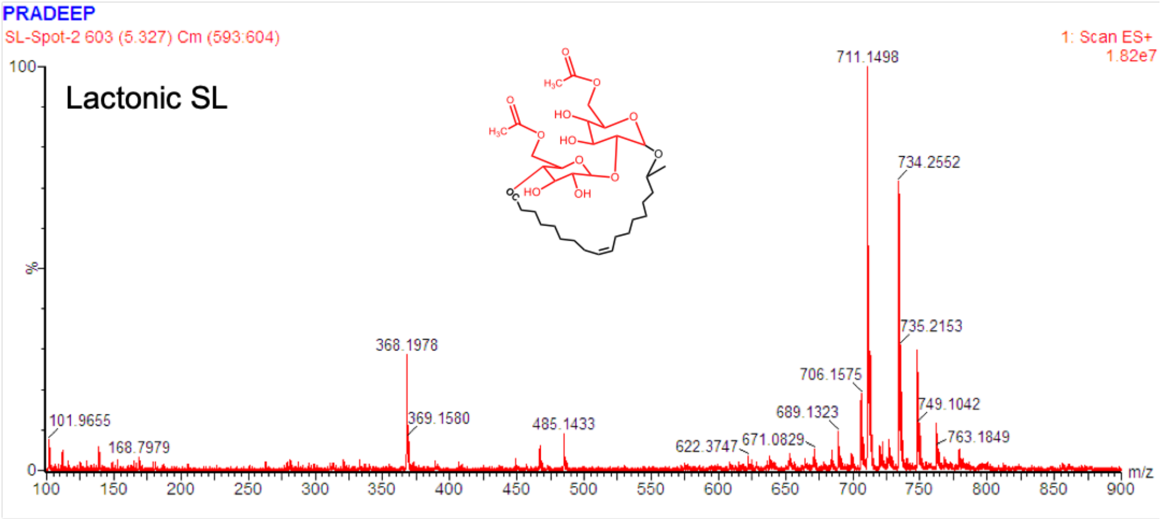

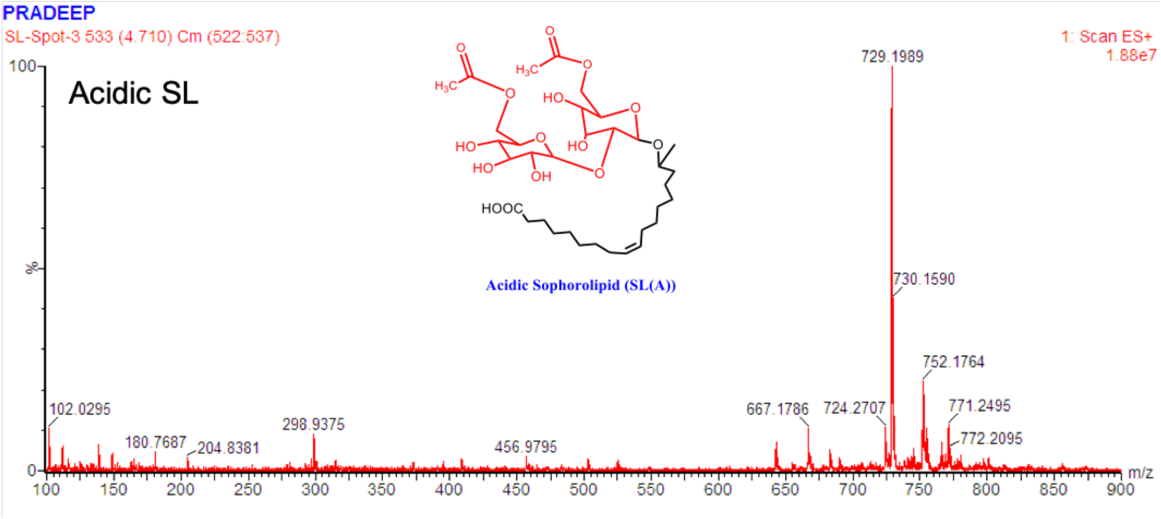

Figure S4. LCMS spectrum of Acidic and Lactonic sophorolipids.

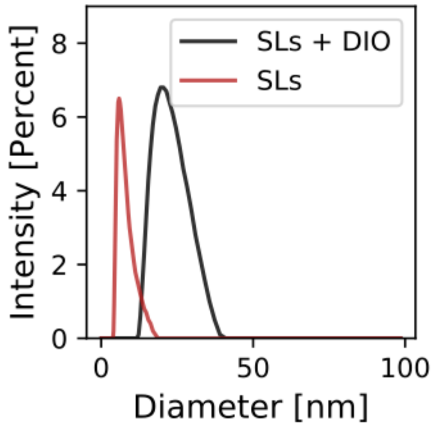

Figure S5. Dynamic Light scattering (DLS) data of sophorolipids micelles (red) and dye encapsulated sophorolipids micelles (black).

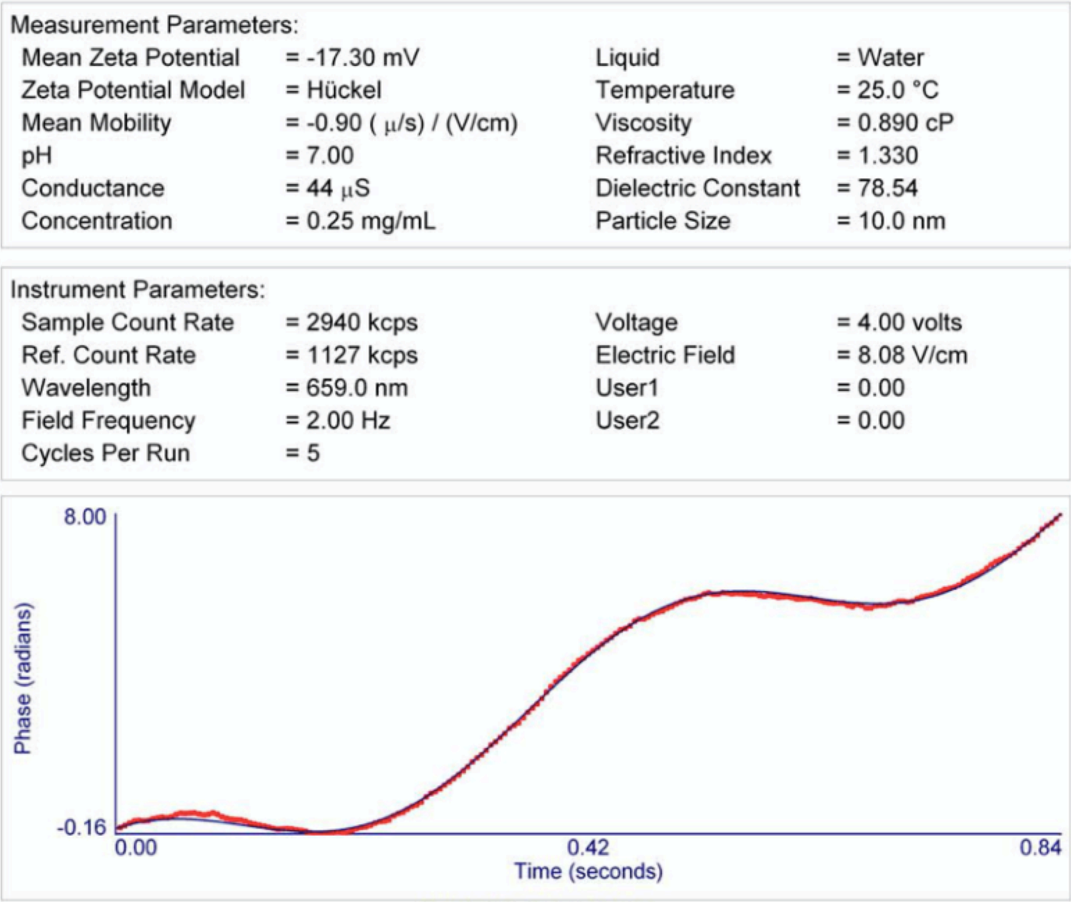

Figure S6. Zeta potential of SL micelles. Note measurements performed at 0.25 mg/ml in water at pH 7.0.

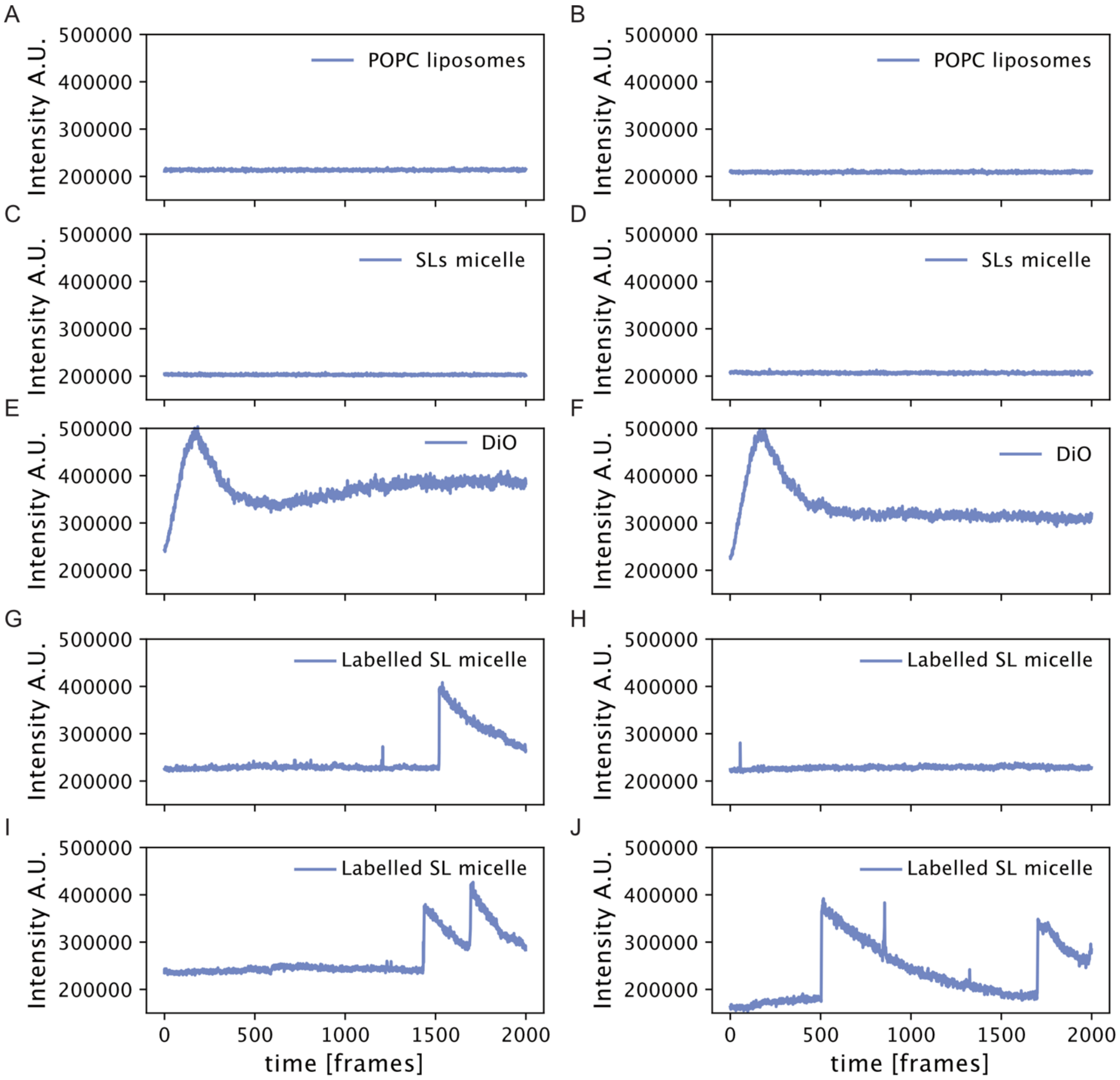

Figure S7: Additional traces and control experiments of SLs micelle docking on surface tethered liposomes. A-B) Free POPC liposomes in solution labelled with DiO. C-D) Non-labelled SLs micelles in solution. E-F) DiO in DMSO added to solution. G) DiO labelled SL micelles docking and fusion. H) As G, but here only displaying kiss'n'run type event. I-J) As G and H, but here displaying multiple docking and subsequent fusion.

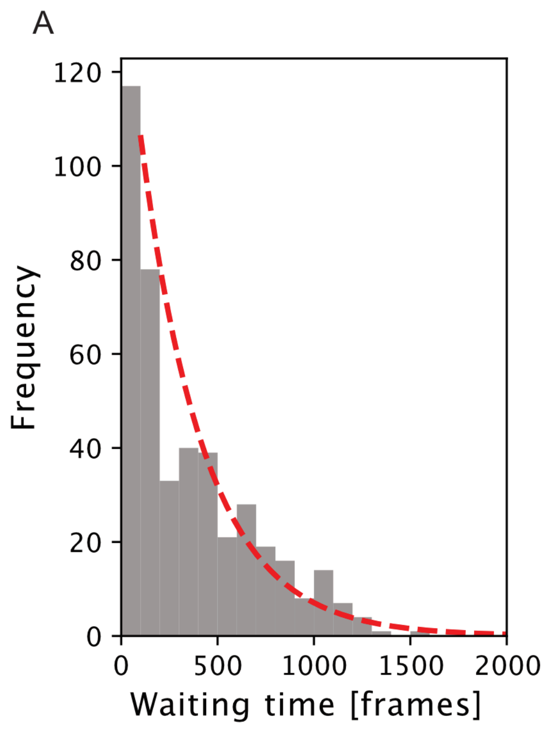

Figure S8. Exponential fitting of consecutive rates. Representative fitting of the waiting time between consecutive rates (here for pH 6.5 and the first consecutive event). A) Histogram of waiting times (grey) and single exponential decay fit (red). Fitting is done using an unbinned likelihood approach (see methods) to avoid potential bias from binning. B) As a, but displayed on a logarithmic y-axis to illustrate the single exponential decay by a straight line.

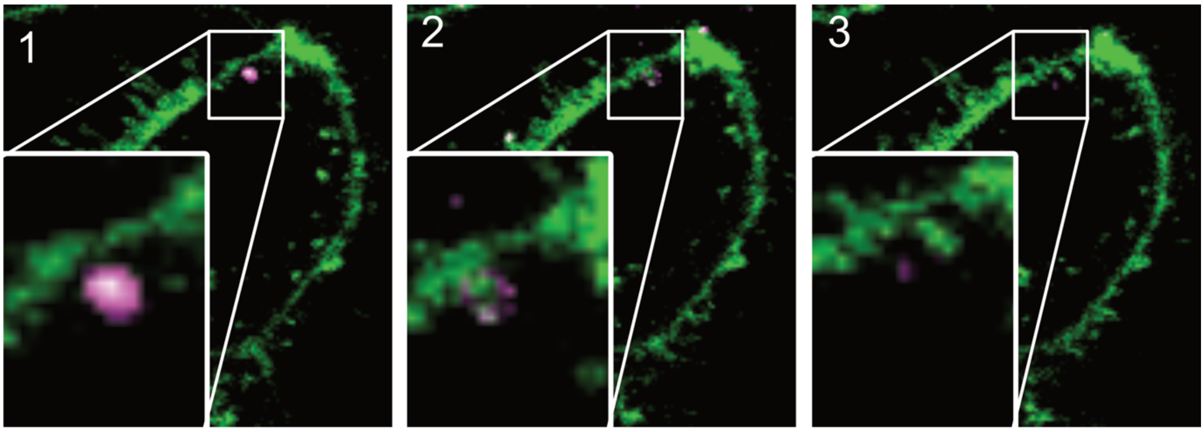

Figure S9. Time lapse of SL micelle on Hela cell displaying a docked particle fusing with the cellular membrane causing fluorescent signal loss. (Time lapse over approximately 10 sec).

Table S1. Individual liposome composition and zeta potential

DOPS (mol %)	DOPC (mol %)	DSPE-PEG(2000)-BIOTIN (mol %)	DSPE-ATTO655 (mol %)	Zeta potential mV*
2	97	0.5	0.5	-3.39 ± 0.47
5	94	0.5	0.5	-4.51 ± 0.21
10	89	0.5	0.5	-7.36 ± 0.69

*Measurements performed in PBS

Table S2. Statistics of all observations measured with three different pH values and three different surface charge.

pH	5.8			6.5			7.4		
SURFACE CHARGE	**-2**	**-5**	**-10**	**-2**	**-5**	**-10**	**-2**	**-5**	**-10**
LIPOSOMES IMAGED	1242	1608	1817	2011	1214	1666	1399	1427	1711
TOTAL EVENTS	902	1164	1192	1388	1903	1315	1370	1495	1427
LIPOSOMES WITH EVENTS	845	1083	1093	1197	1244	1229	1195	1325	1309
LIPOSOMES WITH 1 OR MORE EVENTS	634	816	803	917	818	909	858	954	975
LIPOSOMES WITH 2 OR MORE EVENTS	211	267	290	280	426	320	337	371	334
KISS-AND-RUNS	537	1197	1192	1221	1040	1512	1891	2218	1911
FRACTION OF EVENTS PER LIPSOMES	0,73	0,72	0,66	0,69	1,57	0,79	0,97	1,04	0,83
LIKELIHOOD OF MORE THAN 1 EVENT	0,33	0,32	0,36	0,3	0,52	0,35	0,39	0,39	0,34
